# Molecular Dynamics Simulation of Drug Solubilization Behavior in Surfactant and Cosolvent Injections

**DOI:** 10.3390/pharmaceutics14112366

**Published:** 2022-11-03

**Authors:** Meiqi He, Wenwen Zheng, Nannan Wang, Hanlu Gao, Defang Ouyang, Zunnan Huang

**Affiliations:** 1 The First Dongguan Affiliated Hospital, Guangdong Medical University, Dongguan 523710, China; 2 State Key Laboratory of Quality Research in Chinese Medicine, Institute of Chinese Medical Sciences (ICMS), University of Macau, Macau 999078, China; 3 Key Laboratory of Big Data Mining and Precision Drug Design of Guangdong Medical University, Key Laboratory of Computer-Aided Drug Design of Dongguan City, Key Laboratory for Research and Development of Natural Drugs of Guangdong Province, School of Pharmacy, Guangdong Medical University, Dongguan 523808, China; 4 Department of Clinical Laboratory, The Sixth Affiliated Hospital of Sun Yat-sen University, Guangzhou 510655, China

**Keywords:** solubilization, surfactant, cosolvent, molecular dynamics simulation, water-insoluble drug

## Abstract

Surfactants and cosolvents are often combined to solubilize insoluble drugs in commercially available intravenous formulations to achieve better solubilization. In this study, six marketed parenteral formulations with surfactants and cosolvents were investigated on the aggregation processes of micelles, the structural characterization of micelles, and the properties of solvent using molecular dynamics simulations. The addition of cosolvents resulted in better hydration of the core and palisade regions of micelles and an increase in both radius of gyration (R_g_) and the solvent accessible surface area (SASA), causing a rise in critical micelle concentration (CMC), which hindered the phase separation of micelles. At the same time, the presence of cosolvents disrupted the hydrogen bonding structure of water in solution, increasing the solubility of insoluble medicines. Therefore, the solubilization mechanism of the cosolvent and surfactant mixtures was successfully analyzed by molecular dynamics simulation, which will benefit future formulation development for drug delivery.

## 1. Introduction

Intravenous administration is an essential type of drug delivery that allows the drug to avoid absorption obstacles to enter the circulation directly. New drugs are usually administered intravenously for toxicological evaluation and to obtain basic pharmacokinetic parameters such as volume of distribution, clearance, half-life, and absolute bioavailability during preclinical development [1,2]. With a more extensive utilization of combinatorial chemistry and high-throughput screening technology in drug development, more and more candidate chemicals with high molecular weight, lipid solubility, and low water solubility are entering the research field [3]. More than 40% of the new chemical substances developed by the pharmaceutical industry are poorly water-soluble [3,4,5]. In order to achieve favorable intravenous delivery, the required dosage of the drug should be formulated in a solution-type state that would prevent precipitation at the injection spot from further reduction of the drug in the blood. Thus, drugs with low solubility are a dominant obstacle in the development of intravenous administration [2].

Conventional methods for developing intravenous formulations of practically insoluble drugs include pH modification, addition of cosolvents, micellar solubilization, and complexation of cyclodextrins [6,7,8,9]. The addition of cosolvents is one of the most effective techniques to promote the solubilization of nonpolar drug molecules by reducing the polarity of a large number of solvents more closely to nonpolar solutes [10,11,12]. Cosolvents commonly used in the market for intravenous injections include methanol, ethanol, glycerol, propylene glycol, dimethylacetoamide, and polyethylene glycol 300 [13]. Intravenous formulations solubilized with cosolvents are diluted many times before administration to reduce pain and discomfort at the injection site. Although cosolvent approaches can increase drug solubility and dissolution rates at multiple magnitudes, their success is constrained primarily by toxic effects, especially at high concentrations. In addition, dilution could induce precipitation of drugs due to the exponential relationship between the ratio of cosolvent and the solubility of the solute [14,15].

Surfactant adding is also a technology that can improve the solubility of drugs. Due to their amphiphilic structure, surfactants have been usually employed to dissolve drugs with low water solubility by incorporating them into micelles [16,17,18]. Nonionic surfactants have been more extensively applied to the pharmaceutical field than anionic and cationic surfactants, owing to their high efficacy and low toxicity, including Tween 60, Tween 80, Cremophor EL, and Poloxamer 188 [19]. To maximize solubility and prevent precipitation after dilution, cosolvents are widely used in the market along with surfactants [14,15,20]. For example, Cyclosporine is dissolved by a combination of 65% *v*/*v* polyoxyethylene castor oil 35 and 32.9% *v*/*v* ethanol. It needs to be diluted at least 50–100 times with 0.9% sodium chloride or 5% dextrose solution before administration [21]. However, to our knowledge, the combined solubilizing effect of surfactants and cosolvents on water-insoluble drugs in terms of molecular mechanisms is lacking.

In recent years, along with the massive increment of computational power and the application of efficient computing equipment, Molecular dynamic simulation (MD) has been demonstrated to be a remarkably powerful tool for characterizing and analyzing drug-carrier interactions at the molecular level, and is currently used extensively in the field of pharmaceutical formulation [22,23]. Maleki et al. employed a molecular dynamics simulation approach and discovered that five mer N-isopropyl acrylamide-Carbon nanotube carriers with short polymer chain lengths exhibited optimum interaction with Doxorubicin, which indicated the most desired loading delivery for Doxorubicin [24]. Rezaeisadat et al. used MD to investigate the drug delivery system of PNIPAAm-b-PEG block copolymer solubilizing curcumin molecule, showing that the presence of PNIPAAm-b-PEG polymer increased the solubility of drug by about 88% [25]. Khezri et al. investigated the interaction and release properties of curcumin with chitosan through MD simulations and experimental studies, which revealed the ability of chitosan nanoparticles to carry curcumin [26]. 

In order to improve the solubilized effect of combined solubilization and develop the formulation better, it is of great importance to investigate the mechanism of the combination solubilization of surfactant and cosolvent for intravenous injection. In this study, six intravenous formulations in which the surfactants and cosolvents co-solubilize poorly soluble drugs were collected in the Food and Drug Administration database firstly. The MD simulation was employed to study the above six marketed available parenteral formulations solubilized by surfactants and cosolvents. We built the system using one molecule of the drug and a corresponding number of surfactant and cosolvent molecules based on the molar ratio of drug, surfactant, and cosolvent used in the formulation, as well as the number of water molecules. We mainly analyzed the process of micelle formation, the structural characteristics of micelles, and the properties of solvents to evaluate the solubilizing ability of surfactants and cosolvents. To further analyze the mechanism of the combination solubilization of surfactant and cosolvent, we also established the system without a cosolvent by keeping the number of drug and surfactant molecules unchanged and adding the number of water molecules to replace the removed cosolvent molecules. The mechanism of insoluble drugs solubilized by the combination of surfactant and cosolvent was further revealed by studying the changes in the solubilization process relating to the existence and absence of cosolvent.

## 2. Modeling and Methods

### 2.1. Formulation Information

Six commercially available injectable formulations with co-solubilization of surfactants and cosolvents from the FDA are illustrated in Table 1. Notably, the surfactants used in the six formulations all use the nonionic surfactants Polyoxyl 35 Castor oil or Tween 80. Moreover, ethanol is used as a cosolvent in all formulations. However, Etoposide prescription uses ethanol and polyethylene glycol 300 (PEG 300) for cosolvent solubilization. These injectable formulations are prepared in a non-aqueous preconcentrate form before use and diluted to a particular concentration in a solution medium with 5% dextrose or 0.9% sodium chloride. 

### 2.2. Simulation Details

**Figure 1:**
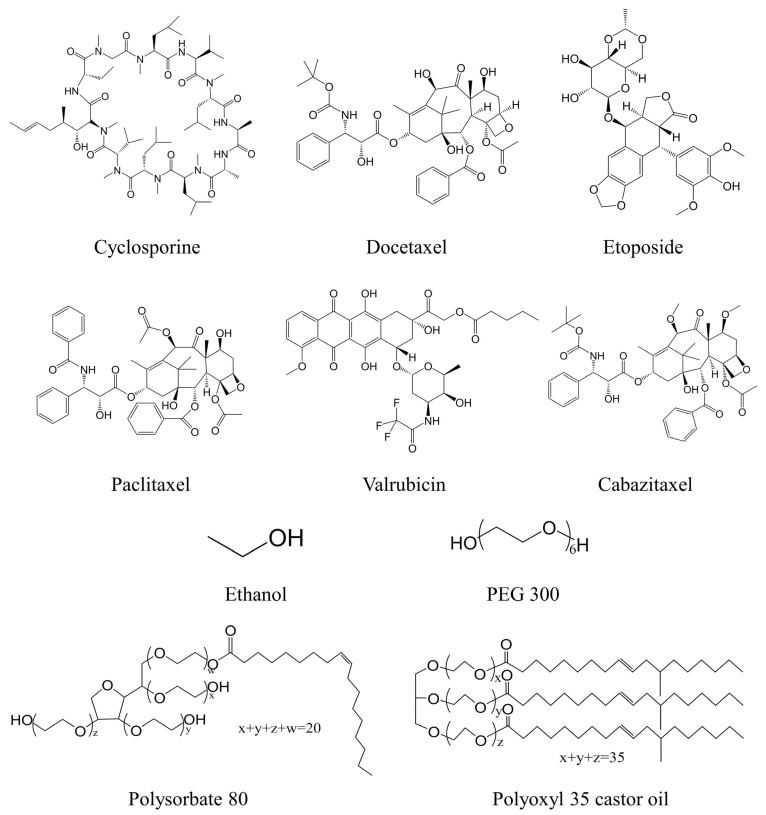
Chemical structures of drugs and excipients.

Chemical structures of 6 drugs (Cyclosporine, Docetaxel, Etoposide, Paclitaxel, Valrubicin, Cabazitaxel), 2 cosolvents (ethanol, PEG 300), and 2 surfactants (polysorbate 80 and polyoxyl 35 castor oil) are illustrated in Figure 1. Molecular types of polysorbate 80 with branched chains of the same length were used (w, x, y, and z = 5) [27]. In addition, the commonly used chain length (x = y = 12, z = 13) was adopted for each polyoxyl 35 castor oil [28]. The models of all drugs and excipients were built and minimized in Discovery Studio 2019 (DS). In the first step of the minimization process, each drug and excipients were minimized with an initial energy using a conjugate gradient algorithm. Then, equilibration was performed at 300 K temperature and NVT ensemble for 100 ps.

In this paper, the molecular dynamics models are set up based on the drug and excipient concentrations of the marketed formulations. To approach a realistic drug delivery environment, the amounts of drug and excipients used in our models are dependent on the molar ratio of drug, surfactant, andcosolvent in the drug formulations. Initial models in the presence and absence of cosolvent containing a single drug and the corresponding number of surfactant molecules in solution were built under periodic boundary conditions. The details of the simulated systems are provided in Table 2. The number of water molecules was set to keep the drug within the clinical use concentration range. 

All simulations were performed using the AMBER 2018 program with general AMBER force field (GAFF) [29]. The TIP3P water model was loaded [30]. The partial atomic charges for drugs and excipients were generated from AM1-BCC. The LEaP, Antechamber, SANDER, and CPPTRAJ modules in the AMBER package were used for preparing MD simulation and analyzing the MD trajectories, respectively. The cut-off distance for the non-bonded interaction was set to 1.0 nm for all systems. The integration time step was set to 2 fs and the SHAKE algorithm was performed to restrain the hydrogen bonds [31]. Then, all systems performed a 5000-step steepest-descent energy minimization followed by a 5000-step conjugate gradient energy minimization. Finally, another 20,000 steps of the steepest-descent energy minimization were carried out for all the systems. Once all models were prepared, the models were subjected to a 20 ps temperature raise to 300 K at NVT ensemble firstly, followed by a 500 ps NPT equilibration at 300 K temperature and 1 atm pressure. In the end, the simulation was run for 150 ns in the NPT ensemble for production. 

## 3. Analyses and Results

### 3.1. Aggregation Processes of Micelles

**Figure 2:**
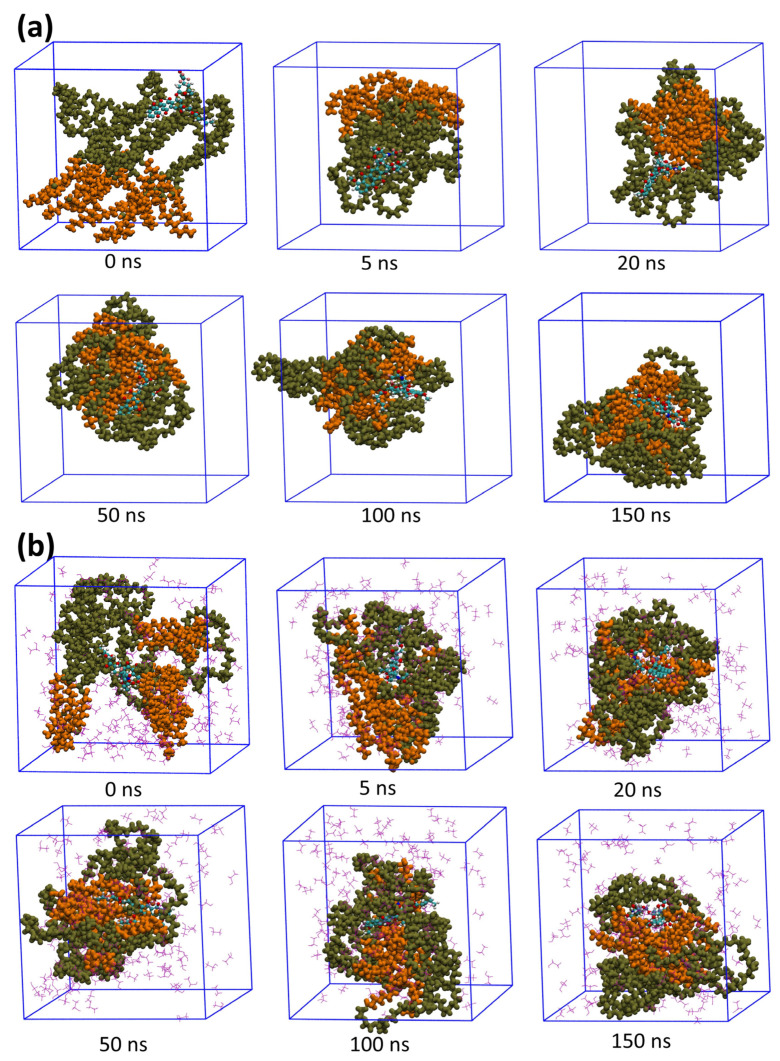
Trajectory snapshots at different simulation times of Valrubicin system (**a**) without cosolvents and (**b**) with cosolvents. The color scheme of atoms: purple lines represent cosolvent molecules; orange balls represent hydrophobic chains of polyoxyethylene 35 castor oil; brown balls represent hydrophilic chains of polyoxyethylene 35 castor oil; red, blue, cyan, and gray balls represent oxygen, carbon, nitrogen, and hydrogen atoms of drug molecules respectively. (Water particles have been removed for clarity).

The self-assembly processes of surfactants and drug encapsulation in different water-media systems could be obtained by analyzing the trajectories of the 150 ns simulations. The micellar solubilization processes of Valrubicin systems containing cosolvents and without cosolvents at different times, for instance, are shown in Figure 2. At 0 ns, the drug, cosolvent, and surfactant molecules were randomly scattered in a solvent box filled with water molecules. Because of the hydrophobicity of drugs, relatively large amounts of surfactants gathered around the drug molecules, forming a larger pre-micelle at 5 ns. Then, as time increased, the clusters became more compact, and the drug molecules continuously rearranged their locations in the micelles until a steady state was achieved. After 150 ns of simulation, the water and cosolvent molecules mixed to fill the box, while the surfactant formed a rough ellipsoid to load the drug molecules. The trajectory processes of systems containing cosolvents and without cosolvents showed a similar solubilization behavior.

**Figure 3:**
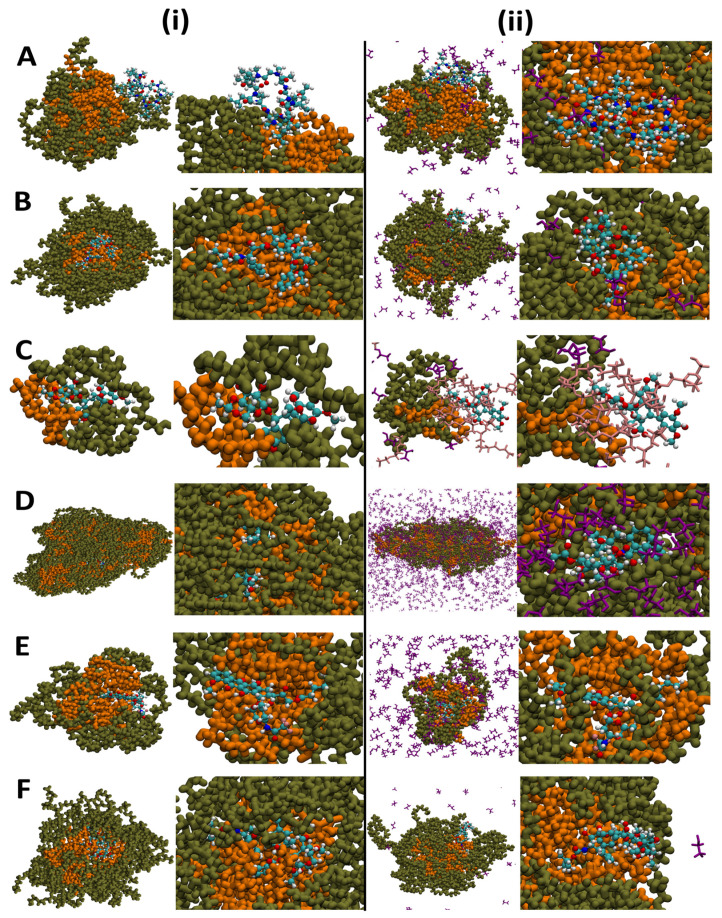
Global and local pictures of the last snapshot in the simulation of (**i**) the systems without cosolvents and (**ii**) the systems with cosolvents. (**A**) represents the Cyclosporine system, (**B**) represents the Docetaxel system, (**C**) represents the Etoposide system, (**D**) represents the Paclitaxel system, (**E**) represents the Valrubicin system, and (**F**) represents the Cabazitaxel system. (Water molecules have been removed for clarity).

The morphological structure of the drug-carrying micelles could be probed by observing the global and local images of the drugs, micelles, and cosolvents in the last frame of the trajectory snapshots, as shown in Figure 3. It could be observed that the surfactants eventually formed a core-shell ellipsoid, with the hydrophilic chains of the surfactants distributed in the outer layer of the micelles, whereas the alkyl groups dominated the core region of the micelles in all systems. Because of the interaction between drug and solvent molecules, drugs were primarily located on the contact surface of the micelles and the solution. Similar results were also obtained for systems without cosolvents. It was intuitively challenging to determine the differences in interaction mode between the systems with and without cosolvents, and further comprehensive analyses should be investigated in the following section.

The ratio of the mean principal axis of inertia (PAI) I_1_:I_2_:I_3_ can be estimated by the mean principal moments of inertia (PMI) over the last 30 ns simulation time to gain the exact morphology of final micelles, which are shown in Table 3. The eccentricity (e) of a micelle is a general measurement of the morphology and magnitude of a micelle and is obtained by the equation $e=\sqrt{1-\frac{c^{2}}{a^{2}}}$, in which c and a represent the shortest and longest semi-axis in the micelle. From Table 3, it can be observed that the eccentricity of the micelles in the simulation varies between the range of 0.63 and 0.96. This indicates that the micelles formed by the surfactant are ellipsoidal spheres, as determined by the eccentricity and the PAI ratio, where a lower eccentricity indicates a more ellipsoidal shape of the micelles.

**Figure 4:**
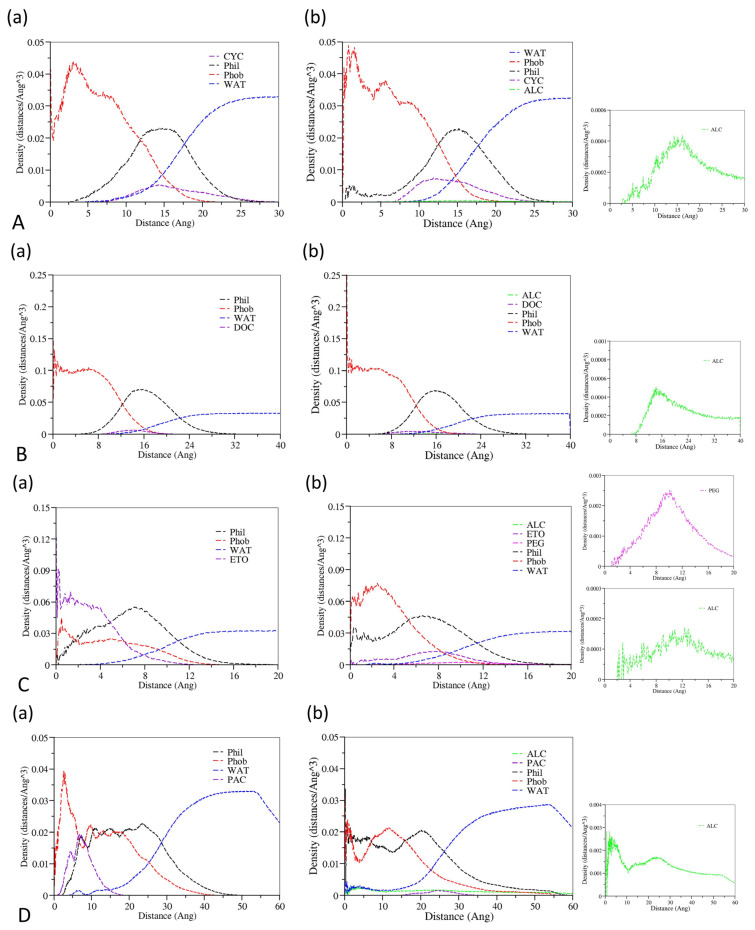

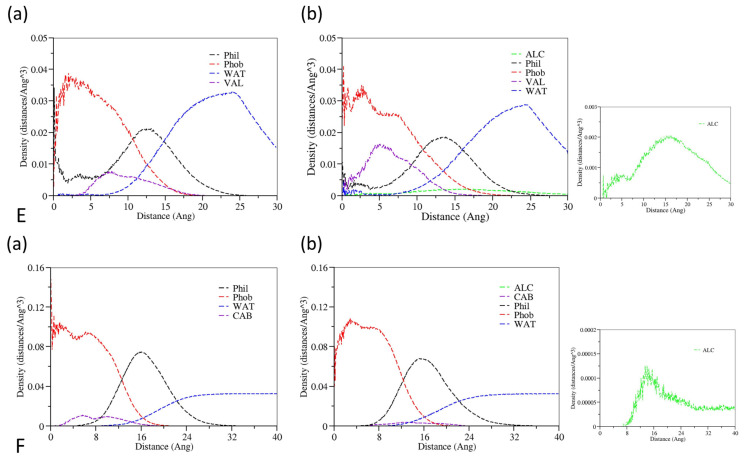
Radial density distributions for various molecular groups against the center core of micelle of (**a**) the formulations without cosolvents and (**b**) the formulations containing cosolvents. (**A**) represents the Cyclosporine system, (**B**) represents the Docetaxel system, (**C**) represents the Etoposide system, (**D**) represents the Paclitaxel system, (**E**) represents the Valrubicin system, and (**F**) represents the Cabazitaxel system. Red represents the hydrophobic groups of the surfactants, black represents the hydrophilic groups of the surfactants, blue represents the water molecules, purple represents the drug molecules, green represents the ethanol molecules, and pink represents PEG 300.

Radial distribution functions (RDFs) could describe how the density of surrounding matters varies as a function of the distance from a point, which could be used to provide insight into the microscopic structure of a drug-loaded micelle. Radial density distribution diagrams for drugs, cosolvents, waters, and the hydrophobic and hydrophilic chains of surfactants as a function of the distance against the center of mass of drug-loading micelles are shown in Figure 4. It could be concluded that hydrophobic groups are primarily distributed in the micellar core, hydrophilic groups are mainly located in the shell layer in all formulations, and drug molecules are dominantly soluble in the micellar core-shell structure. At the same time, the drug molecules also have some contact with the water phase. In addition, the water molecules are mainly distributed in the solution phase, and a small number of water molecules will contact the micellar core. In the distribution range of hydrophilic chains of surfactants, cosolvents have a certain amount of distribution concentration, which indicates that a small fraction of cosolvent molecules permeate into the micelles and interact with hydrophilic chain molecules. Except for the Paclitaxel formulation, there were no significant differences in the micellar internal structure between the systems containing cosolvents and without cosolvents. The RDF of the Paclitaxel system comprising cosolvents showed that hydrophilic groups and hydrophobic groups tend to be located in the core of the micelle; however, this is due to the limitations of RDF analysis methods. The eccentricity of Paclitaxel micelles in the system with cosolvents was 0.96 and formed a relatively flat ellipsoid according to the analysis of the last frame diagram and eccentricity described above, so the radial density distribution diagram could not exactly explain its structure.

**Figure 5:**
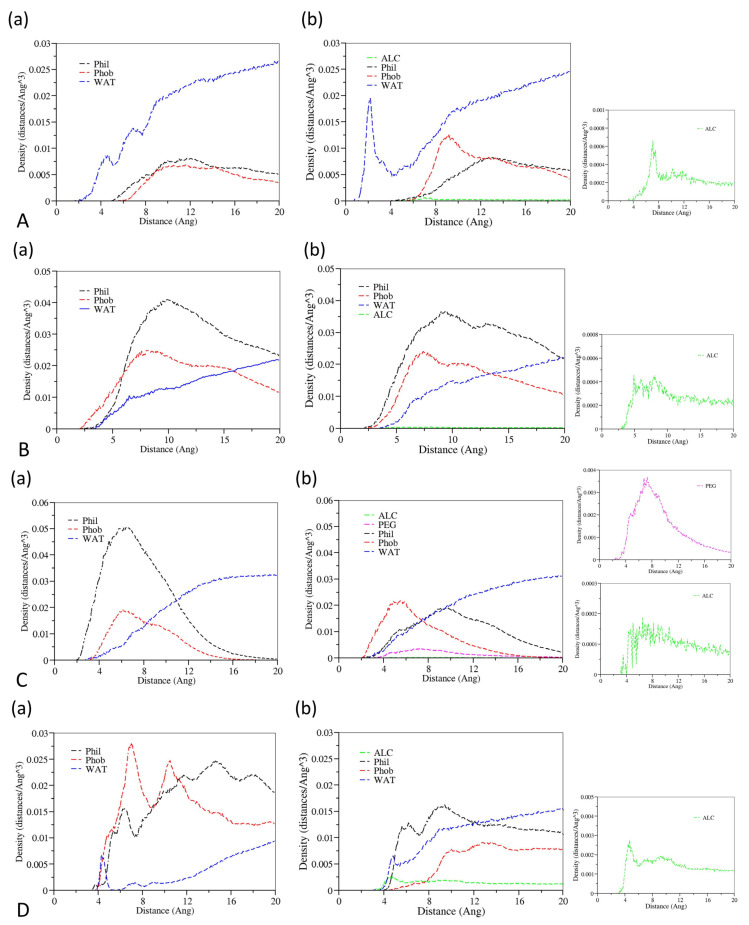

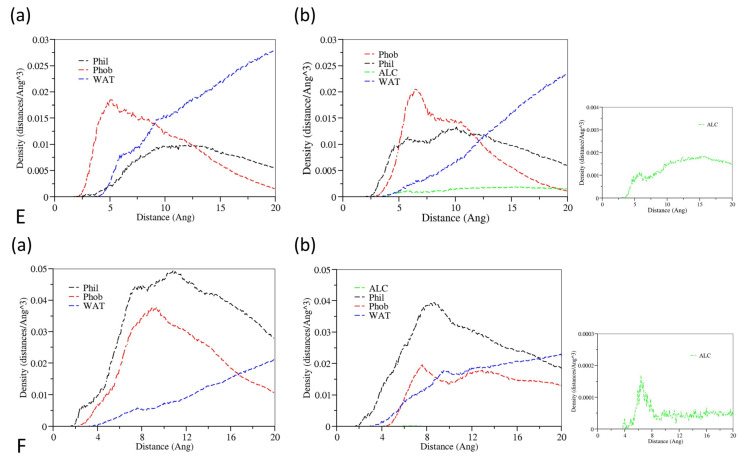
Radial density distributions for various molecular groups against the center of drug particles of (**a**) the formulations without cosolvents and (**b**) the formulations containing cosolvents. (**A**) represents the Cyclosporine system, (**B**) represents the Docetaxel system, (**C**) represents the Etoposide system, (**D**) represents the Paclitaxel system, (**E**) represents the Valrubicin system, and (**F**) represents the Cabazitaxel system. Red represents the hydrophobic groups of the surfactants, black represents the hydrophilic groups of the surfactants, blue represents the water molecules, purple represents the drug molecules, green represents the ethanol molecules, and pink represents PEG 300.

By depicting the location and local environment of the drug molecules in the carrier environment, the interactions of drugs with excipients can be derived. Figure 5 shows the RDFs for water particles, cosolvents, and hydrophobic and hydrophilic groups against drug particles at the solubilized state in the presence and absence of cosolvents. From these analyses, we could conclude that drug molecules mainly solubilized in the interface of hydrophobic and hydrophilic groups, where drug molecules interacted with water molecules and cosolvents at the same time. In addition, cosolvent molecules gathered around drug molecules within 4–8 angstroms, indicating that drug molecules and cosolvent molecules have a certain affinity. The Etoposide molecule was gathered by a considerable volume of PEG 300 chains and ethanol chains under the solubilization of two cosolvents [24,25].

### 3.2. The Effect of Cosolvent on the Properties of Bulk Solvent

Under the concept of solubility, a nonpolar solute in aqueous solution would be limited by the ordered water structure around the nonpolar moieties, which would restrict the solubility of the substance with low solubility [32,33]. It has been reported that the existence of some cosolvents, for example, ethanol, propanol and DMSO, could act as structure breakers, leading to the breakdown of the hydrogen bonding structure of water around hydrocarbon substances, weakening the hydrophobic interaction between the nonpolar group and water molecules and thus increasing drug solubility [34,35]. Therefore, it is crucial to study the properties of the bulk solution after adding the cosolvents, since changes in the solution properties would indirectly impact the solubility of insoluble solutes and surfactants [36,37].

**Figure 6:**
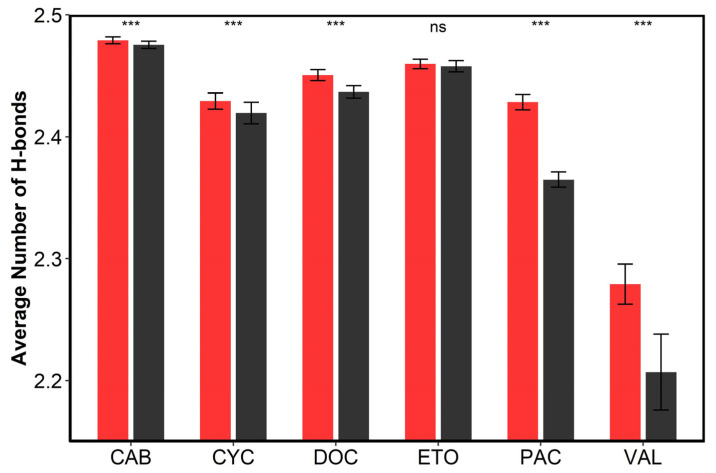
The number of hydrogen bonds between water−water (per water) in formulations without cosolvents (red column) and the formulations containing cosolvents (black column). Error bars are plotted as ±SD. The symbol “***” represents statistical significance with *p* < 0.001, and the symbol “ns” represents no statistical significance.

Based on the above analysis, the ethanol molecules added to the micellar solution were dominantly distributed in the bulk solution, with a small number of molecules penetrating the micelles to interact with surfactants. The hydrogen bonding number (per water) between water–water for all systems is plotted in Figure 6. The differences in the number of water molecule–water molecule hydrogen bonds formed per water molecule between the systems without cosolvents and those with cosolvents were analyzed using the Student’s *t*-test and the Mann–Whitney U test by R software (version 4.1.0, R Foundation, Vienna, Austria), as shown in Table S1. Regarding the effect of cosolvents on the hydrogen bonding number of water–water, we observed that their values decreased after the addition of the cosolvents for all systems except for the Etoposide system, which is particularly obvious for the Paclitaxel and Valrubicin systems. It could be associated with the differences in the concentrations of ethanol added to the different systems. The concentrations of ethanol added to the Paclitaxel and Valrubicin systems were about 7.61% *w*/*w* and 10.27% *w*/*w*, respectively, which were much larger than those of the Docetaxel (1.42% *w*/*w*), Cyclosporine (1.31% *w*/*w*), Etoposide (0.52% *w*/*w*), and Cabazitaxel (0.27% *w*/*w*) systems. Because ethanol molecules could form hydrogen bonds with water, a number of water–water hydrogen bonds were replaced by water–ethanol hydrogen bonds. An ethanol molecule could not replace a water molecule to create a tetrahedron hydrogen bond network since an ethanol molecule contains only one hydrogen bond, whereas a water molecule contains two. Therefore, the original hydrogen bonding network of pure water molecules was destroyed, and the average hydrogen bonds formed by each water molecule were reduced [38]. As for the Etoposide system, the cosolvents formulated consist of ethanol and PEG 300. The PEG 300 fails to break the water hydrogen bonding network as ethanol does, despite being miscible with water [39]. Since the the octanol and water partition coefficients (log P) of PEG 300 is close to −1.93, which is closer to the log P of ethanol (−2.4) compared to water, it is possible for a part of the alcohol molecule to be adsorbed on PEG 300, leading to a reduction in the distribution of alcohol in the solution. 

**Figure 7:**
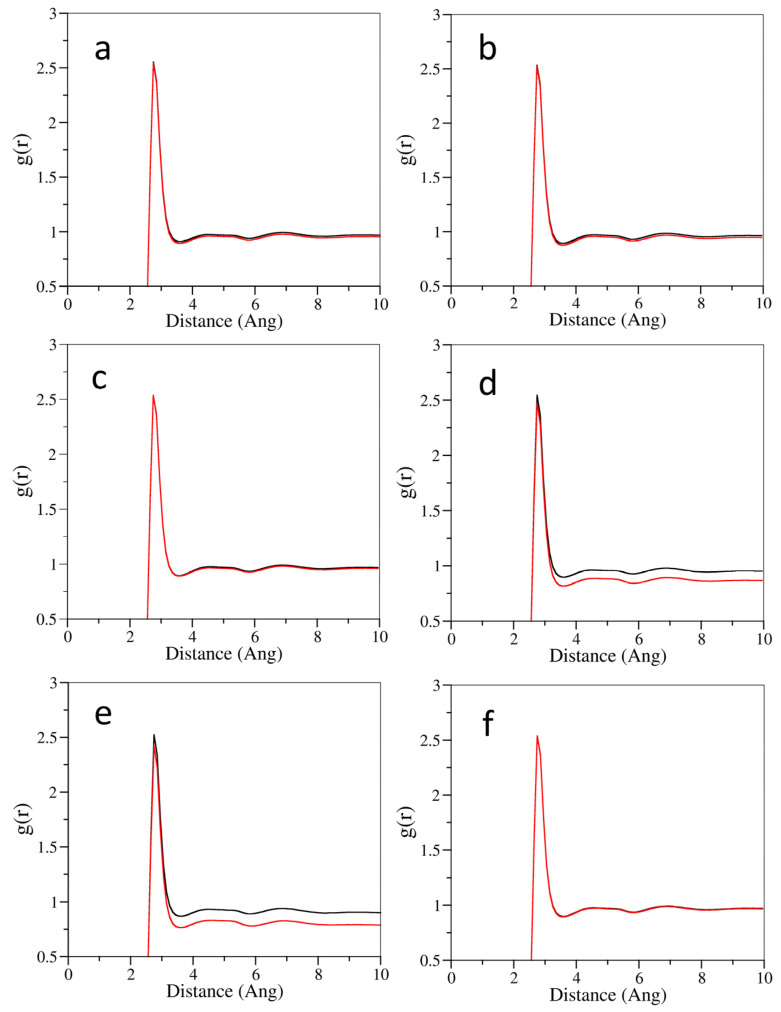
The radial distribution functions for water oxygen–water oxygen (O_w_–O_w_) sites of formulations without cosolvents (dark line) and the formulations containing cosolvents (red line). (**a**) represents the Cyclosporine system, (**b**) represents the Docetaxel system, (**c**) represents the Etoposide system, (**d**) represents the Paclitaxel system, (**e**) represents the Valrubicin system, and (**f**) represents the Cabazitaxel system.

To investigate the effect of cosolvents on the water structure, the radial distribution functions of the water-oxygen–water-oxygen (O_w_–O_w_) sites for all simulations are shown in Figure 7. The first peak of the O_w_–O_w_ site distribution function at 2.75 corresponded to the first adjacent hydrogen bond on the tetrahedron. The first peak of the O_w_–O_w_ distribution function overlapped with the system without the cosolvent molecule after adding cosolvents, however, the height showed an insignificant decrease. The overall O_w_–O_w_ height became shallower with respect to the system without cosolvents after the first peak. It was obvious that the number of water–water hydrogen bonds developed in the system containing cosolvents was reduced, and the water hydrogen bonding network was interrupted compared to the system without cosolvents. The addition of cosolvents promoted the solubility of nonpolar solutes by interfering with the hydrogen bonding network of aqueous solutions, thereby reducing the structure of water molecules near nonpolar hydrocarbon groups [33]. The hydrophilic hydrogen bonding groups of most cosolvents ensured miscibility with water, whereas their hydrophobic hydrocarbon regions interfered with the hydrogen bonding network of water, reducing the hydrogen bond density of water and lowering the chemical potential of the solution, thereby providing a less polar environment to attract more drug molecules into the solution [32].

### 3.3. The Effect of Cosolvent on Micellar Formation

In addition to modifying the properties of the overall solution phase and weakening the hydrophobic effect, the presence of cosolvents could also affect the micelle formation by surfactant molecules, thus affecting the micellar solubilization capacity.

**Figure 8:**
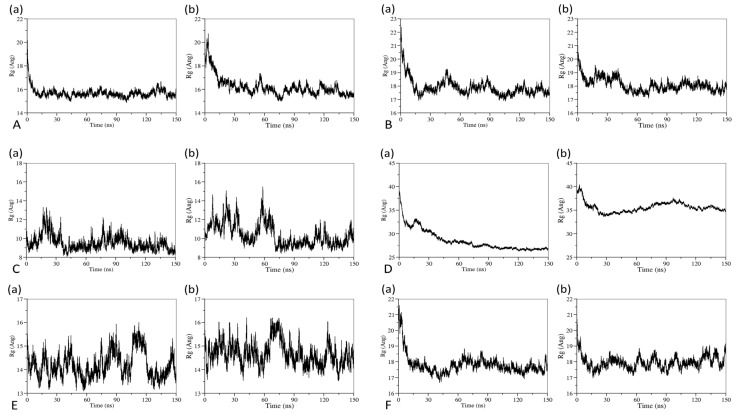
R_g_ evolutions during the simulation time of (**a**) the systems without cosolvents and (**b**) the systems with cosolvents. (**A**) represents the Cyclosporine system, (**B**) represents the Docetaxel system, (**C**) represents the Etoposide system, (**D**) represents the Paclitaxel system, (**E**) represents the Valrubicin system, and (**F**) represents the Cabazitaxel system.

**Figure 9:**
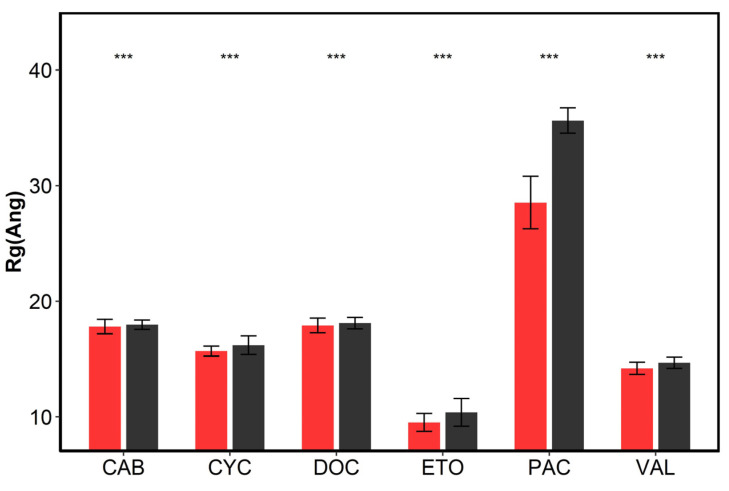
The average R_g_ values of different systems without cosolvents (red column) and containing cosolvents (black column) during the last 30 ns simulation time. Error bars are plotted as ±SD. The symbol “***” represents statistical significance with *p* < 0.001.

The variation of radius of gyration (R_g_) of drug-loaded micelles throughout simulation time was used as a factor to indicate the collapsed structure of micelles. Figure 8 shows the time variations of R_g_ of drug-loaded micelles in the systems with and without cosolvents during the 150 ns simulation. As it could be seen, the R_g_ of micelles became smaller and smaller over time until they reached a stable state and fluctuated around a certain value in both systems with and without cosolvents, which indicated that micellar aggregates became more and more compact. This also shows that the simulation time we used is sufficient to reach equilibrium. In addition, the average R_g_ during the last 30 ns of the simulation time was shown to characterize the size of micelles in Figure 9. The Student’s *t*-test and Mann–Whitney U test in R software (version 4.1.0, R Foundation, Vienna, Austria) were adopted to analyze the differences of the R_g_ during the last 30 ns between the systems with cosolvents and without cosolvents, as shown in Table S2. It could be found that the R_g_ of systems solubilized by surfactants and cosolvents were larger than systems without cosolvents. The impact of the addition of cosolvents on the micellar structure should be further analyzed.

**Figure 10:**
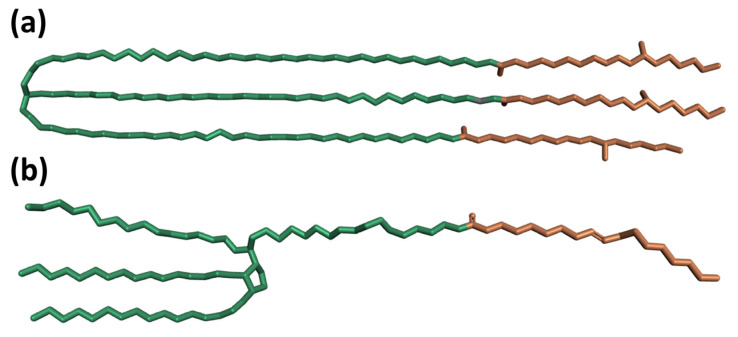
The hydrophilic (green) and hydrophobic (orange) chains of (**a**) Polyoxyl 35 castor oil and (**b**) Polysorbate 80.

**Figure 11:**
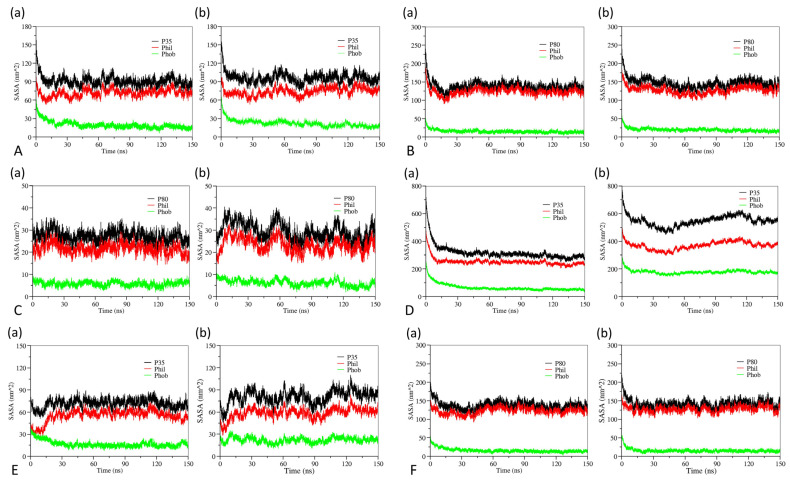
The time-evolution of the solvent accessible surface area (SASA) of (**a**) the formulations without cosolvents and (**b**) the formulations containing cosolvents. (**A**) represents the Cyclosporine system, (**B**) represents the Docetaxel system, (**C**) represents the Etoposide system, (**D**) represents the Paclitaxel system, (**E**) represents the Valrubicin system, and (**F**) represents the Cabazitaxel system.

The surface area of biomolecules that could be contacted by solvents was called the solvent accessible surface area (SASA), which was an analytical method to measure the solubility of insoluble substances [40]. In order to investigate the variation of micellar structure more intensively, the polyethylene oxide (PEO) chains are classified as hydrophilic chains and the other hydrocarbon moieties as hydrophobic chains (Figure 10). To quantify the hydrophilicity of micelles, we calculated the time evolutions of SASA of all surfactants in formulations and their hydrophilic groups and hydrophobic groups over the simulation time, as shown in Figure 11. It could be seen from the figure that the SASA of surfactants and their hydrophilic ends and hydrophobic groups in all systems increased or decreased and finally reached equilibrium at a certain value. In addition, the SASA of the hydrophilic chains of all the surfactants was more significant than the SASA of the hydrophobic chains, indicating that surfactants gradually formed a stable micelle with hydrophilic chains distributing in the outer layer and hydrophobic groups gathering at the core. Moreover, the average SASA of surfactants, hydrophilic groups, and hydrophobic chains were calculated to characterize the micelle structure during the last 30 ns time, as shown in Table 4. Hydrophobic contribution parameters (hydrophobic%) as the ratio of SASA of hydrophobic chains to the total SASA of the last 30 ns time were also displayed in Table 4. The SASA of micelles in formulations containing cosolvents was more significant than that of corresponding formulations without cosolvents. It was consistent with the comparative analysis of R_g_ mentioned above. Except for the Etoposide formulation, the SASA of their hydrophobic chains, hydrophilic groups, and hydrophobic% in all formulations containing cosolvents were more significant than that of corresponding formulations without cosolvents. These indicated that the affinity of micelles formed by surfactants and solution was relatively larger in the presence of cosolvents, which indicated the addition of ethanol resulted in swelling (higher degree of solvation) of both the core and corona. Moreover, it also revealed that the hydrophobic chains of surfactants in the systems containing cosolvents interacted more with the solvent molecules, which may lead to changes in the distribution of the micelle structure. The Etoposide formulation was slightly different because its cosolvents were composed of ethanol and PEG 300. The average SASA of hydrophobic chains in the Etoposide formulation containing cosolvents was 4.7982 square nm, whereas the average SASA of hydrophobic chains without cosolvents was 5.7641 square nm. The latter has a larger hydrophobic surface area because the hydrophilic and hydrophobic chains of micelles competed to bind the PEG 300 chains. However, both hydrophilic chains and PEG 300 have longer polyoxyethylene segments [-CH2-CH2-O-] in their structures, and the octanol and water partition coefficients (log P) for both near ethylene glycol is –1.93, indicating that PEG 300 has a greater affinity for hydrophilic chains. Therefore, PEG 300 had more interaction with the hydrophilic chain than with the hydrophobic chain, resulting in a restricted activity interval for the hydrophobic chain. 

### 3.4. The Interactions between Drugs and Excipients

**Figure 12:**
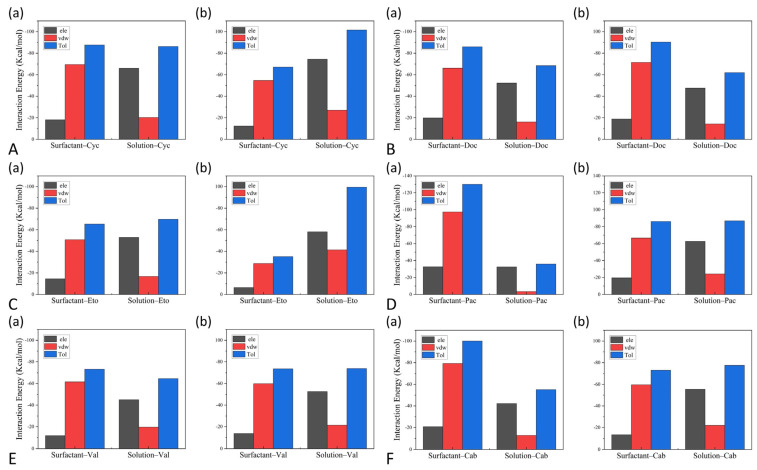
Interaction energies of each binary component of (**a**) the formulations without cosolvents and (**b**) the formulations with cosolvents, where ele stands for the electrostatic interaction energy and vdw refers to the van der Waals interaction energy. (**A**) represents the Cyclosporine system, (**B**) represents the Docetaxel system, (**C**) represents the Etoposide system, (**D**) represents the Paclitaxel system, E represents the Valrubicin system, and F represents the Cabazitaxel system.

The interactions between micellar chains, solution particles, and drug molecules were analyzed to characterize the solubility variation of the drug after co-solubilization by surfactants and cosolvents, as shown in Figure 12. In this regard, the solution molecules comprise water molecules and cosolvent molecules in the solution phase. The van der Waals interactions between the drugs and the micelles are more significant than the electrostatic interactions because fewer groups in the micellar chains are capable of forming hydrogen bonds with the drug particles. In contrast, electrostatic interactions between drugs and solvent molecules are more prominent than van der Waals interactions owing to the presence of a large number of -O-H that can form hydrogen bonds between water molecules. However, few hydrogen bonds are formed between drug and water molecules. As observed in Figure 12, the interaction between drug particles and solvent molecules was more robust in the system containing cosolvents than in the system without cosolvents, except for the Docetaxel system. The addition of ethanol rendered the aqueous solution more favorable for dissolving the drug. It increased the solvation of the micelles with reduced contact with the drugs, resulting in a greater affinity of the drug molecules to the solution. It is indicated that the micelles would reduce the encapsulation of drug particles after adding cosolvents.

When the cosolvents were added to the aqueous solution of the surfactants, the R_g_ and SASA of the micelles increased, indicating an increase in the solvation of the hydrophilic and hydrophobic chains of the micelles, which was related to the change of properties of the solution. As the hydrogen bonding network of the aqueous solution was broken after the addition of the cosolvents, the disruption of the water structure around the nonpolar groups made the hydrophobic interaction of the hydrophobic chains weaker. Since the hydrophobic interaction generated by the hydrophobic tail is the main driving force for micelle formation, the addition of ethanol hindered the binding and stability of micelles, leading to an increase in the critical micelle concentration, which is detrimental to the formation of micelles. Furthermore, ethanol molecules were distributed mainly in the solution and penetrated less into the micelles when added to the solution, so the effect of ethanol on the formation of micelles through this mechanism will be limited. Moreover, the addition of ethanol molecules diminishes the encapsulation capacity of micelles. There are several experimental studies that correspond to this finding. Alexandridis et al. added ethanol molecules into the solution of block copolymer Pluronic P105, and the solvent content in both the micelle core and corona increased [34]. Kawakami et al. observed that the solubility of the Tween 80 solution increased when ethanol was added [41]. In the face of dilution, surfactants with a higher critical micelle concentration (CMC) are more likely to face the risk of drug precipitation and lower solubilization ability to solubilize drugs.

## 4. Discussion

Nonionic surfactants and ethanol are widely used in surfactant and cosolvent co-solubilized injections. These injectable formulations are prepared in a non-aqueous preconcentrate form before use and are diluted to a particular concentration in a solution medium. The molecular dynamics simulation study showed that the surfactants formed ellipsoidal micelles rapidly after dilution and were wrapped insoluble drugs between the hydrophilic and hydrophobic chains of the micelles. Only a small fraction of cosolvent and water molecules penetrated into the micelles and mostly mixed in a homogeneous solvent phase. However, the research mainly focused on nonionic surfactants and ethanol, and the lack of research on ionic surfactants and other cosolvents will lead to the lack of study of overall excipients and relevant information on the application of formulations. In the future, we plan to extend this analysis to the formulation applications of other surfactants, cosolvents, and insoluble drugs.

The drug exhibits linear solubilization in surfactant solution and follows a log-linear model in cosolvent solution [41,42]. However, the combined solubilization of surfactants and cosolvents depends on the interaction of surfactant and cosolvent and is not simply a superposition. In general, in the case of surfactant and cosolvent solubilization, a portion of the cosolvent molecules is distributed in the bulk solution, and part of the cosolvent molecules penetrates into the micelle to interact with surfactants. In this work, with the addition of ethanol to the solution, most of the ethanol molecules were distributed in the solution, which changed the properties of the solution and disrupted the hydrogen bonding network of the aqueous solution, making the solution less polar and more suitable for the solubilization of insoluble drugs and surfactants. However, the decreased hydrophobic interaction of surfactants hindered the formation of micelles, which decreased micelle stability and affected the solubilization of drugs in micelles. Therefore, the solubilization capacity of insoluble drugs in surfactants and cosolvents is difficult to be directly generalized empirically and needs to be obtained by analytically observing the combined interaction of the cosolvent and surfactant. In this paper, molecular dynamics simulation was adopted to study the co-solubilization mode of surfactants and cosolvents, which could reveal the interaction between the cosolvent and the surfactant from a microscopic molecular perspective; therefore, it is of great significance to use molecular dynamics simulation for the research of drug delivery.

## 5. Conclusions

Our MD simulation study provided a molecular perspective for understanding the solubilization behavior of nonpolar drugs in systems solubilized by surfactants and cosolvents. We could conclude that randomly dispersed surfactant molecules self-assembled into ellipsoidal micelle with a core of hydrophobic groups and an outer layer of hydrophilic groups encapsulating poorly water-soluble drugs. It was found that drug particles were preferentially localized at the core–shell interface of micelles, where they interacted with water, cosolvent molecules, and hydrophobic and hydrophilic groups. A small fraction of water and cosolvent molecules were inserted into the outer shell of the micelles. It could also be concluded that the addition of ethanol increased the solvation of the core and palisade regions of the micelles, which contributed to the rise in the CMC of the micelles. At the same time, ethanol increased the solubility of insoluble drugs in solution due to the disruption of the hydrogen bonding network of water molecules. Therefore, the molecular mechanism of cosolvent and surfactant co-solubilization was successfully explored by molecular dynamics simulations, which is expected to play a central role in the future in the drug delivery field. In the future, we may be able to use molecular dynamics simulation to screen the excipients for the combined solubilization approach of surfactants and cosolvents.

## Figures and Tables

**Table 1:** Market formulations solubilized by the combination of surfactants and cosolvents.

Brand Name/Generic Name	Formulation (Each mL)	Clinical Use
Sandimmune/Cyclosporine	Cyclosporine 50 mg Polyoxyl 35 castor oil 650 mg Ethanol 32.9% (*v*/*v*)	20 mL solution diluted with 100 mL Sodium Chloride or Dextrose injection
Taxotere/Docetaxel	Docetaxel 20 mg Polysorbate 80 540 mg Ethanol 395 mg	Diluted with Sodium Chloride or Dextrose injection to 0.3–0.74 mg/mL
Toposar/Etoposide	Etoposide 20 mg Citric acid 2 mg Polysorbate 80 80 mg Polyethylene glycol 300 650 mg Ethanol 262 mg	Diluted with Dextrose or Sodium Chloride Injection to 0.2–0.4 mg/mL
Taxol/Paclitaxel	Paclitaxel 6 mg Polyoxyl 35 castor oil 527 mg Ethanol 49.7% (*v*/*v*) Citric acid 2 mg	Diluted with Sodium Chloride or Dextrose injection to 0.3–1.2 mg/mL
Valstar/Valrubicin	Valrubicin 40 mg Polyoxyl 35 castor oil 50% *v*/*v* Ethanol 50% *v*/*v*	20 mL Valstar diluted with 55 mL Sodium Chloride Injection
Jevtana/Cabazitaxel	Cabazitaxel 40 mg Polysorbate 80 1040 mg	Diluted with 5.7 mL 13% (*w*/*w*) ethanol solution, followed by dilution of sodium chloride or dextrose solution

**Table 2:** The details of the simulated systems.

System	NO. of Molecule(s)				Cdrug ^3^ (mg/mL)	Cdrug ^4^ (mg/mL)
	Drug	Surfactant	Cosolvent	Water		
Cyc ^1^	1	6	137	25,502	0.5–2.5	2.45
Doc ^1^	1	17	346	60,026	0.3–0.74	0.70
Eto ^1^	1	2	68/167 ^5^	80,258	0.2–0.4	0.40
Pac ^1^	1	30	1228	33,933	0.3–1.2	1.12
Val ^1^	1	4	157	2917	10.67	10.04
Cab ^1^	1	17	183	160,932	0.1–0.26	0.26
Cyc ^2^	1	6	-	25,927	-	2.45
Doc ^2^	1	17	-	61,091	-	0.70
Eto ^2^	1	2	-	81,794	-	0.40
Pac ^2^	1	30	-	37,622	-	1.12
Val ^2^	1	4	-	3388	-	10.04
Cab ^2^	1	17	-	161,486	-	0.26

^1^ The system containing cosolvents, ^2^ The system without cosolvents, ^3^ The clinical concentration of drugs in collected formulations, ^4^ The final concentration of drug used in the simulations, ^5^ The system contains 68 PEG 300 molecules and 167 ethanol molecules.

**Table 3:** The PAI ratio and eccentricity of the simulated systems.

**Complex**	**Cyc ^1^**	**Cyc ^2^**	**Doc ^1^**	**Doc ^2^**	**Eto ^1^**	**Eto ^2^**
I_1_:I_2_:I_3_	1.37:1.26:1	1.43:1.26:1	1.34:1.18:1	1.30:1.18:1	1.76:1.51:1	1.81:1.55:1
e	0.69	0.71	0.67	0.64	0.82	0.83
**Complex**	**Pac ^1^**	**Pac** **^2^**	**Val ^1^**	**Val ^2^**	**Cab ^1^**	**Cab ^2^**
I_1_:I_2_:I_3_	1.68:1.49:1	3.62:3.52:1	1.59:1.34:1	1.49:1.33:1	1.28:1.17:1	1.46:1.28:1
e	0.80	0.96	0.78	0.74	0.63	0.73

^1^ The system without cosolvents, ^2^ The system containing cosolvents.

**Table 4:** The R_g_ and SASA values of systems without cosolvents and with cosolvents.

**System**	**Cyc**	**Cyc-co**	**Doc**	**Doc-co**	**Eto**	**Eto-co**
SASA(nm^2^) ^1^	90.25	95.36	135.89	147.02	26.23	27.12
SASA(phob) ^2^	16.22	17.88	12.92	16.13	5.76	4.80
SASA(phil) ^3^	74.03	77.48	122.97	130.89	20.47	22.32
hydrophobic % ^4^	17.97	18.75	9.51	10.97	21.97	17.69
**System**	**Pac**	**Pac-co**	**Val**	**Val-co**	**Cab**	**Cab-co**
SASA(nm^2^) ^1^	284.55	574.53	69.21	90.31	135.24	143.44
SASA(phob) ^2^	51.29	176.93	14.96	23.95	12.73	13.81
SASA(phil) ^3^	233.26	397.60	54.25	66.36	122.51	129.63
hydrophobic% ^4^	18.02	30.80	21.62	26.52	9.41	9.63

^1^ The average SASA of all surfactants, ^2^ The average SASA of the hydrophobic chains of all the surfactants, ^3^ The average SASA of the hydrophilic chains of all the surfactants, ^4^ Hydrophobic contribution parameters.

## Data Availability

All related data and methods are presented in this paper. Additional inquiries should be addressed to the corresponding author.

## Supplementary Materials

The following supporting information can be downloaded at: https://www.mdpi.com/article/10.3390/pharmaceutics14112366/s1, Table S1: The significance test between the number of hydrogen bonds between water−water (per water) in formulations without cosolvents and the formulations containing cosolvents. Table S2: The significance test between the average R_g_ values of different systems without cosolvents and containing cosolvents during the last 30 ns simulation time.
