# Supplementary material for: Molecular Dynamics Simulation of Drug Solubilization Behavior in Surfactant and Cosolvent Injections
Source: Pharmaceutics. 2022 Nov 3;14(11):2366. doi: 10.3390/pharmaceutics14112366 (PMC9692798; doi:10.3390/pharmaceutics14112366)
Supplement: Supplementary file 1 [file pharmaceutics-14-02366-s001.zip › pharmaceutics-1933625-supplementary.pdf]

pharmaceutics

MDPI

Supplementary Materials

Molecular Dynamics Simulation of Drug Solubilization Behavior in Surfactant and Cosolvent Injections

Meiqi He, Wenwen Zheng, Nannan Wang, Hanlu Gao, Defang Ouyang and Zunnan Huang

Table S1. The significance test between the number of hydrogen bonds between water−water (per water) in formulations without cosolvents and the formulations containing cosolvents.

Systems	$\overline{x}$ ± S	95% CI	*P* (Student's *t*-Test)	*P* (Mann–Whitney *U* Test)
Cyc [1]	2.4292+_0.0066	0.0058~0.0139	<0.001	<0.001
Cyc [2]	2.4194+_0.0089			
Doc [1]	2.4505+_0.0046	0.0113~0.0163	<0.001	<0.001
Doc [2]	2.4367+_0.0050			
Eto [1]	2.4597+_0.0039	−0.0003~0.0041	0.093	0.050
Eto [2]	2.4578+_0.0046			
Pac [1]	2.4284+_0.0064	0.0602~0.0668	<0.001	<0.001
Pac [2]	2.3649+_0.0062			
Val [1]	2.2791+_0.0166	0.0591~0.0851	<0.001	<0.001
Val [2]	2.2070+_0.0311			
Cab [1]	2.4790+_0.0029	0.0023~0.0053	<0.001	<0.001
Cab [2]	2.4752+_0.0029			

[1] The system without cosolvents. [2] The system with cosolvents.

Table S2. The significance test between the average R_g values of different systems without cosolvents and containing cosolvents during the last 30 ns simulation time.

Systems	$\overline{x}$ ± S	95% CI	*P* (Student's *t*-Test)	*P* (Mann–Whitney *U* Test)
Cyc [1]	15.68 ± 0.43	−0.53~−0.50	<0.001	<0.001
Cyc [2]	16.20 ± 0.80			
Doc [1]	17.90 ± 0.65	−0.22~−0.19	<0.001	<0.001
Doc [2]	18.10 ± 0.50			
Eto [1]	9.52 ± 0.78	−0.90~−0.84	<0.001	<0.001
Eto [2]	10.38 ± 1.20			
Pac [1]	28.53 ± 2.26	−7.13~−7.05	<0.001	<0.001
Pac [2]	35.62 ± 1.10			
Val [1]	14.19 ± 0.54	−0.50~−0.48	<0.001	<0.001
Val [2]	14.68 ± 0.49			
Cab [1]	17.80 ± 0.63	−0.17~−0.15	<0.001	<0.001
Cab [2]	17.96 ± 0.42			

[1] The system without cosolvents. [2] The system with cosolvents.
